# Systematic Review of Clinical Prediction Models for the Risk of Emergency Caesarean Births

**DOI:** 10.1111/1471-0528.17948

**Published:** 2024-09-10

**Authors:** Alexandra Hunt, Laura Bonnett, Jon Heron, Michael Lawton, Gemma Clayton, Gordon Smith, Jane Norman, Louise Kenny, Deborah Lawlor, Abi Merriel, the Options Study Collaborative Group, Sheelagh McGuiness, Anna Davies, Dame Tina Lavender, Christy Burden, Jonathan Ives, Simon Grant, Sherif Abdel‐Fattah, Danya Bakhbakhi, Andrew Demetri, Mairead Black, Sam Finnikin, Amie Wilson, Alexandra Freeman, Pete Blair, Kate Birchenall, Joanne Johnson, Amber Marshall

**Affiliations:** ^1^ Department of Health Data Science The University of Liverpool Liverpool UK; ^2^ Bristol Medical School The University of Bristol Bristol UK; ^3^ Bristol Population Health Science Institute The University of Bristol Bristol UK; ^4^ Department of Obstetrics and Gynaecology The University of Cambridge Cambridge UK; ^5^ The Rosie Hospital Cambridge UK; ^6^ The University of Nottingham Nottingham UK; ^7^ Department of Women's and Children's Health, Faculty of Health and Life Sciences The University of Liverpool Liverpool UK; ^8^ Centre for Women's Health Research, Department of Women's and Children's Health University of Liverpool Liverpool UK; ^9^ Liverpool Women's Hospital Liverpool UK

**Keywords:** emergency caesareans, maternal Health, prediction, prognostic, risk factors

## Abstract

**Background:**

Globally, caesarean births (CB), including emergency caesareans births (EmCB), are rising. It is estimated that nearly a third of all births will be CB by 2030.

**Objectives:**

Identify and summarise the results from studies developing and validating prognostic multivariable models predicting the risk of EmCBs. Ultimately understanding the accuracy of their development, and whether they are operationalised for use in routine clinical practice.

**Search Strategy:**

Studies were identified using databases: MEDLINE, CINAHL, Cochrane Central and Scopus with a search strategy tailored to models predicting EmCBs.

**Selection Criteria:**

Prospective studies developing and validating clinical prediction models, with two or more covariates, to predict risk of EmCB.

**Data Collection and Analysis:**

Data were extracted onto a proforma using the Prediction model Risk Of Bias ASsessment Tool (PROBAST).

**Results:**

In total, 8083 studies resulted in 56 unique prediction modelling studies and seven validating studies, with a total of 121 different predictors. Frequently occurring predictors included maternal height, maternal age, parity, BMI and gestational age. PROBAST highlighted 33 studies with low overall bias, and these all internally validated their model. Thirteen studies externally validated; only eight of these were graded an overall low risk of bias. Six models offered applications that could be readily used, but only one provided enough time to offer a planned caesarean birth (pCB). These well‐refined models have not been recalibrated since development. Only one model, developed in a relatively low‐risk population, with data collected a decade ago, remains useful at 36 weeks for arranging a pCB.

**Conclusion:**

To improve personalised clinical conversations, there is a pressing need for a model that accurately predicts the timely risk of an EmCB for women across diverse clinical backgrounds.

**Trial Registration:** PROSPERO registration number: CRD42023384439.

## Abstract 2

**Linked article**: This article is commented on by Eldamanhoury pp. 241–242 in this issue. To view this article visit https://doi.org/10.1111/1471‐0528.17992.

## 1 Introduction

Globally, the prevalence of caesarean births, including emergency caesarean births (EmCB), continues to rise, whilst the rate of this rise varies between different settings [1], it is an important area of global focus. The National Institute for Health and Care Excellence (NICE) now suggests a planned caesarean birth (pCB) as a safe and positive alternative to vaginal birth (VB), especially if having up to two CB, albeit with different risks and benefits [2]. Other countries facilitate pCB, and in Brazil for example, the CB rate is over 50% [3]. In England specifically, rates of pCB have reached 17%, with 12% of nulliparous women electing for a pCB in 2022–2023.

With many women now able to choose a pCB, it is important to turn attention to EmCBs which are more risky and expensive than pCB, with increased rates of bleeding, infection, surgical complications and fetal trauma, and adverse effects in subsequent pregnancies [4, 5]. Furthermore, EmCB can be psychologically traumatising with up to 50% experiencing stress related symptoms 2 months after caesarean [6]. Rates for EmCB are rising in England 2022–2023, 22% of births were EmCB with 29% of nulliparous women now having an EmCB, and similar trends are seen in many other European countries. For example, data from 2019 showed 27 European countries with a CB rate above 20% [7].

Most EmCBs are performed for those who have opted for a VB [8]; therefore, it is vital women have the opportunity for a more personalised discussion surrounding their birthing options so they can make the best choice for them [8]. This may be particularly important for nulliparous women, for whom we don't have the key predictor of prior births on which to base these discussions.

Choices about mode of birth are influenced by personal, social and family experiences, alongside local cultural norms and values. Current evidence suggests that being in control of decisions can enhance experience of birth [9]. Providing women with more personalised risk information could empower them to better understand their options. This approach may increase pCBs and reduce the rate of EmCB, or it may encourage a woman to opt for a VB where she was considering a pCB, if she is predicted to have a higher chance of success.

Multiple tools to predict EmCBs exist, but they are not yet routinely used. A key element of them being applied in clinical practice is that we have time to act upon the risk of the outcome in clinical context. We aimed to identify the available prediction models which include nulliparous women globally, and understand the robustness of their development, and whether they are operationalised for use in routine clinical practice.

## 2 Methods

This review was prospectively registered on PROSPERO (CRD42023384439; 27.04.2023) and was conducted according to the PRIMSA guidelines [10].

We identified studies developing and validating multivariable prediction models for EmCB by searching bibliographic databases (Medline, Cochrane Central, SCOPUS and CINAHL) from inception to 9 March 2023 (date of search). A prediction model search filter [11] was combined with search terms for caesarean births (see sample Medline search in Data S1).

### 2.1 Inclusion Criteria

Deduplicated searches were uploaded onto the online systematic reviewing software Covidence [12] and further deduplicated. Title and abstracts were initially screened for relevance using the predefined inclusion/exclusion criteria (Table 1) by two independent reviewers (AH and AM). Full texts of studies seeming to meet the inclusion criteria or where there was any uncertainty were retrieved for review.

**TABLE 1:** Inclusion/exclusion.

Inclusion criteria	Exclusion criteria
Multivariable prediction model of emergency caesarean births developed or validated. Population included nulliparous women (could include multiparous women too). Prospective and retrospective cohort study designs. Cohort, case–control and randomised control trials in which (potential) predictors were assessed. Studies of any language or publication. (Translations will be requested)	Studies without the relevant outcome, emergency caesarean births. Studies limited to twin pregnancies. Studies including multiparous women only. Studies not presenting multivariable prediction models (two or more predictors). Reviews of studies (not presenting original data). Abstract and protocols of studies, without a fully published text. Simulation studies not using real data.

### 2.2 Data Extraction

Data extraction was conducted independently onto a piloted data extraction form, created using Google Forms [13]. Disagreements, in regard to eligibility, were resolved through discussion and consultation with a third author. Extraction covered study and patient characteristics, candidate and prognostic factors, frequency and handling of missing data, outcome measures, statistical methods. Detail on model development, any internal or external validation performance statistics were also documented. This included discrimination (c‐statistics or area under the curve) and calibration (expected/observed events ratio). Model information regarding the timing of predictor assessments, timing of the prediction, predictive accuracy and ease of use of the models assisted in determining the model's current clinical utility.

### 2.3 Assessment of Study Quality

Included studies were assessed for risk of bias (quality) using the Prediction model Risk Of Bias ASsessment Tool (PROBAST) [14]. PROBAST guide has 20 signalling questions across four domains: participants, predictors, outcome and analysis. Each signalling question assesses different risk aspects for the corresponding domain. Domains were each graded as a low, moderate or high risk of bias based on the number of questions that introduced bias. Each domain is then assessed, and studies presenting a high risk of bias for any one of these domains were graded as an overall high risk of bias. Furthermore, as per PROBAST guidelines, high risk of bias was ascribed if a study failed to internally and/or externally validate the developed model even if they were low‐risk of bias in the signalling questions. The review extracted information to make an informed decision on bias introduced into a model, guided by PROBAST's domains and signalling questions and focused the predictive accuracy of models deemed as introducing a low risk of bias.

### 2.4 Data Synthesis

We conducted a narrative synthesis of the literature to identify and compare the main findings, strengths and limitations of the identified studies. Information on similarities and differences between studies participants, methods, analysis and results was important. The Preferred Reporting Items for Systematic reviews and Meta‐Analyses (PRISMA) was utilised to provide a checklist and flow diagram, ensuring transparency throughout the review [10]. Conclusion on risk of bias was drawn through PROBAST and clinical utility assessed on the models' performance and use. Certain information has been tabulated, and some visually displayed. We anticipated that a meta‐analysis would not be possible due to heterogeneity.

## 3 Results

**FIGURE 1:**
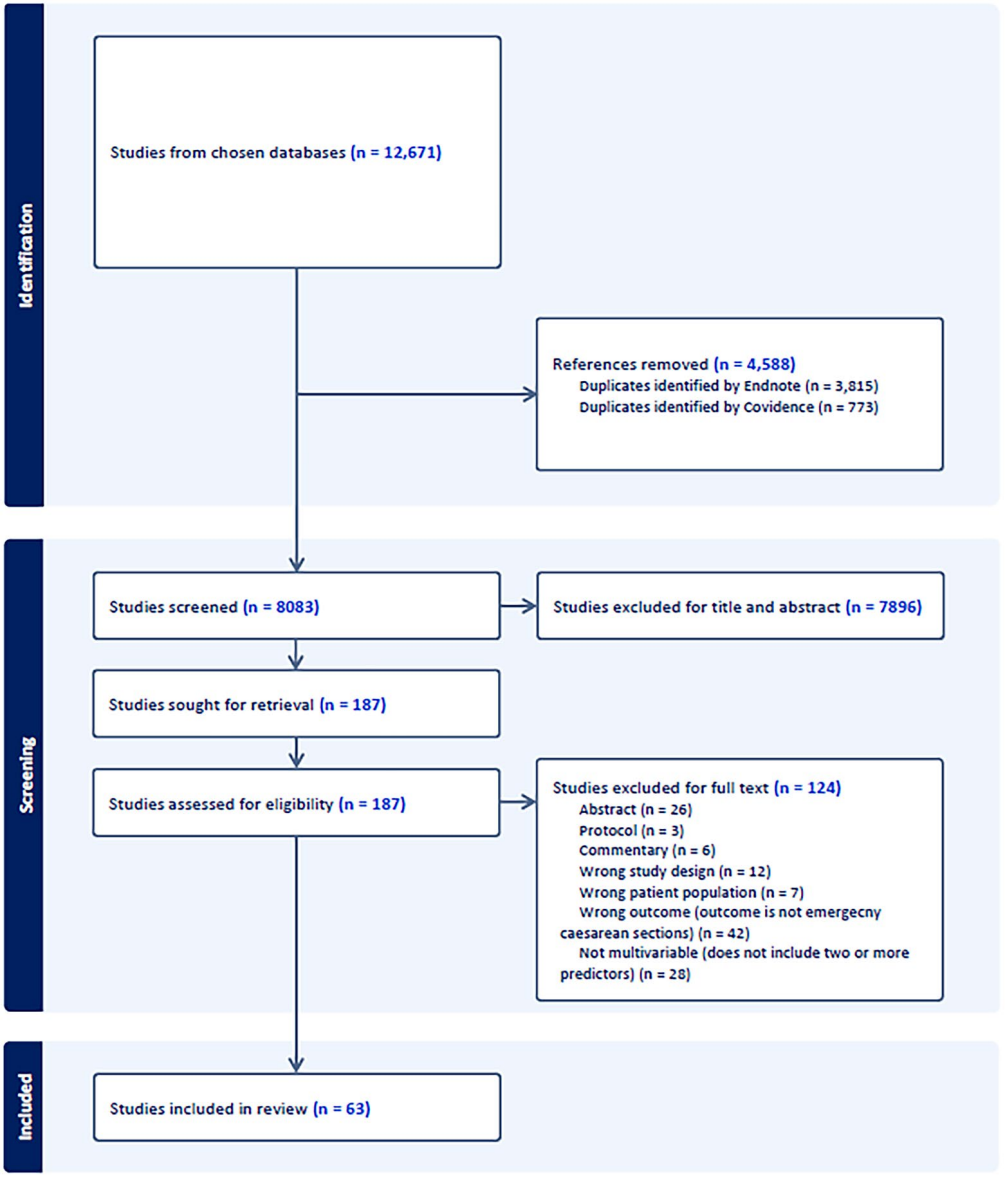
PRISMA flow diagram.

The search yielded 12 671 results across the four databases. After de‐duplication, there were 8083 remaining title and abstracts, of which 63 fulfilled the inclusion criteria. Details are presented in the PRISMA diagram in Figure 1.

The 63 studies included data on 4 476 307 women. Fifty‐six studies reported the development of new prediction models, whilst seven reported the validation of an existing model. These studies were carried out in multiple countries, spanning 15 high income countries and 11 in low‐middle income countries. They were mainly concentrated in the United States (*n* = 11) and China (*n* = 7), as summarised in Figure S1.

Study populations varied. Seventeen studies compromised a population of nulliparous women and 46 presented a mixed population of nulliparous and multiparous women. Fifty‐five studies included spontaneous and induced labours; 23 studies focused on induced labours, 28 on spontaneous labours and four studies considered both induced and spontaneous labours. Eight studies did not provide this information. Across the 63 studies, 14 studies defined the outcome of EmCBs due to a specific indication, rather than encompassing all indications. For example, EmCS due to arrest of labour (*n* = 8), fetal distress (*n* = 1), cephalopelvic disproportion (*n* = 3), macrosomia (*n* = 1) and premature rupture of the membrane (PRoM; *n* = 1).

80.9% (*n* = 51) of the studies developed models based on data generated prior to 2018.

The types of model development identified in the review were logistic regression (44), generalised linear mixed modelling (*n* = 4), partial linear mixed modelling (*n* = 1), unclear methods (*n* = 2) and machine learning techniques [12], such as Adaptive Boosting, Artificial Neural Networks, Classification Algorithms, Decision Trees, Gradient Boosted Trees, Random Forests and Support Vector Machines.

### 3.1 Included Predictors

In total, 121 different model predictors were identified and 24 of these occurred more than twice within different models. Table 2 displays the 24 model predictors that appeared more than twice within the 63 identified models. A full list of frequency of all predictors identified can be found in Table S1.

**TABLE 2:** The 24 model predictors that appeared more than twice across the 63 studies.

Predictor (occurring more than twice across all models)	Occurrences
Maternal height	32
Maternal age	31
Parity	26
BMI	22
Gestational age	18
Bishops score	9
Previous caesarean sections	9
Cervical dilatation	7
Estimated fetal weight	7
Maternal weight	7
Fetal AC	6
Maternal weight gain	
New born weight	6
Station	6
Indication for induction	6
Cervical length	5
Fetal gender	5
Maternal race	5
Previous vaginal birth	5
Synthesis fundal height region	4
Hypertension	4
Pre‐existing diabetes	4
Oxytocin use	3
Type of induction	3

### 3.2 PROBAST Quality Assessment

The PROBAST assessment for the 63 studies, and each PROBAST domain, can be found in Table S2. Thirty‐three studies were graded as introducing low risk of bias and 30 as high risk of bias.

#### 3.2.1 Participants

The population was defined in terms of spontaneous/induced labour in all but eight studies. Low risk of bias was ascribed to retrospective (*n* = 32) and prospective (*n* = 23) observational study designs, and randomised controlled trials (*n* = 2). However, the four included case–control studies did not appropriately adjust for the original cohort or registry outcome frequency and therefore were classed as high risk of bias. Low risk of bias was assigned to 43 studies providing clear and appropriate inclusion and exclusion criteria. The remaining 20 were graded as high risk due to failing to present any exclusion criteria (*n* = 15) or unclear and inappropriate criteria choice (*n* = 5).

#### 3.2.2 Predictors

The definition and measurement of predictors for models must be clear and constant for all participants in the study and models must present predictors available at the time of intended use.

An average of seven predictors (range 2–21) was included in each prediction model. Forty‐seven studies defined all predictors, or the predictors were standard measures. However, 16 studies included undefined predictors without additional supplementary material.

Twenty‐three studies failed to present predictors available for when the model was intended to be used; thus, we graded these as introducing a risk of bias. For instance, four studies included baby's birth weight as a predictor for a model intended to be used before childbirth. One study failed to present this information, falling into the studies presenting inaccessible predictors.

Overall 13 studies presented bias regarding how studies defined, measured or selected predictors.

#### 3.2.3 Outcome

Choosing to perform an EmCS can be necessary due to multiple reasons, including maternal request. Studies with outcomes for EmCSs based on specific complications were carefully determined. Studies mostly used a generic outcome of all EmCS (*n* = 48), but 15 studies presented specific EmCS due to several conditions such as arrest of labour (*n* = 8), fetal distress (*n* = 1), cephalopelvic disproportion (*n* = 3) and macrosomia (*n* = 1).

All 63 studies presented appropriate outcome definitions.

#### 3.2.4 Analysis

Analysis methods significantly impact bias risk. Studies presenting a low risk of bias will have appropriate use of statistical analysis methods, such as model stability, handling of predictors, performance measures, missing data and validation techniques. Omission of statistical considerations increase bias potential.

##### 3.2.4.1 Model Stability

Fifty‐six studies developed a new prediction model. Sample size varied from *n* = 97 to *n* = 4 177 644. The PROBAST guidelines refer to the number of events per variable (EPV = number of EmCBs divided by the number of levels of all candidate predictors), within this review EPV varied from 1.54 to 18 614.5; however, more than half did not reach commonly cited threshold of EPV greater than 10 [15, 16], although some evidence suggests 50 might be more appropriate [17, 18]. Twenty‐three studies calculated an EPV greater than 10 and of these eight exceeded an EPV of 50. EPV was not calculable in 15 studies due to unclear candidate predictor parameters.

Studies validating an existing model (*n* = 7) consisted of sample sizes varying from *n* = 240 to *n* = 6 530 467. Validation studies aim to transparently quantify the predictive accuracy of an existing model; therefore, these studies must present the methods to calculate the EPV and/or direct the reader to any supplementary material presenting this information. Three of the seven validation studies presented the information needed to calculate the EPV. We deemed the remaining four studies as introducing risk of bias.

##### 3.2.4.2 Handling Predictors

Continuous predictors are optimal when not dichotomised [19]; 38 studies with well‐described handling were low‐risk, whilst 25 studies, with five introducing bias through dichotomisation and 20 inadequately explaining cut‐off methods, were high risk.

The methods used to select predictors is extremely important. Among 43 studies, 18 used backwards stepwise, a statistically preferred method; 17 used forwards stepwise; and eight used machine learning, presented no bias risk. Thirteen studies had unclear methods.

##### 3.2.4.3 Performance Measures

Discrimination and calibration serve as appropriate indicators for evaluating a prognostic prediction model's predictive accuracy, representing the most suitable metrics for assessing its performance and thus, done properly pose no risk of bias. Discrimination describes the model's ability to distinguish between those who experienced an EmCB and those who did not. Usually measured using the concordance statistic, known as Harrell's C‐statistic, discrimination provides a scale ranging from 0.5 (no better than chance) to 1 [20]. Sixty studies presented discrimination in the form of a C‐statistics or graphically as a ROC curve, offering a visual assessment. Calibration refers to the measures of how well the predicted probability aligns with the number of actual EmCBs. This is assessed graphically using a calibration slope, providing a visual estimation, alongside a calibration value. Twenty studies presented calibration plots.

##### 3.2.4.4 Missing Data

Missing data can also sometimes occur and with appropriate handling, such as multiple imputation, the risk of bias, although still present, can be significantly reduced.

Three studies used multiple imputation to counteract data that were missing at random (MAR). Multiple imputation uses existing data to replicate plausible values and imputed the missing data for that variable, completing this multiple times and pooling results across all completed datasets, allowing the use of larger datasets whilst incorporating uncertainty in imputed values [21]. Twenty‐seven studies described excluding participants with missing data, known as complete case analysis. Approaches using complete case analysis or single imputation potentially introduce bias. The remaining studies failed to appropriately describe the handling of the missing data. According to PROBAST guidelines, these studies flag potential for bias because information is missing.

##### 3.2.4.5 Validation

Of the studies developing a model, 33 studies included a form of internal validation, whilst 13 externally validated the developed model. Methodology for internal validation included the statistically preferred bootstrap resampling in 16 studies and cross‐validation in four studies.

Fifty studies presented the models weighted regression coefficients with confidence intervals, six without confidence intervals and only 36 provided an intercept to the logistic regression. In 31 studies, a discrepancy between the final model and reported multivariable analysis results, without explanation or re‐estimation, was deemed high bias risk. Mismatches may arise if predictors are excluded without re‐estimating all coefficients.

Overall, the PROBAST assessed only two studies as a high risk of bias within their analyses.

#### 3.2.5 PROBAST Conclusion

Considering the four risks of bias domains together, 30 studies were rated as a high risk of bias and 33 as low risk of bias. Nineteen studies were graded as a high risk of bias due to an overall high risk across one of the four domains, and the further 11 studies were downgraded from a moderate risk to a high risk due to no internal validation.

Eight studies [22, 23, 24, 25, 26, 27, 28, 29], considered as a low risk, externally validated their developed model with an independent dataset. Only these eight models could be considered for use in a clinical setting subject to further external validation and a thorough assessment of clinical utility. All eight studies employed logistic regression for model development, with four focusing on nulliparous women and four on a mixed population. One study [25] aimed to predict EmCB for macrosomia, whilst the other seven predicted all EmCBs. One model [22] intended prediction at 39–40 weeks and another at 36 weeks [26], the remaining six predicted risk pre‐induction. Although a prediction pre‐induction may be useful for clinicians, it provides little time for the mother to have a discussion on her birthing options.

The eight studies presented 23 different predictors within their models and the top 3 frequently occurring predictors were maternal height, maternal age and gestational age (Table 3).

**TABLE 3:** Model predictors identified from the studies assessed as low risk of bias with external validation.

Predictors	Burke [22]	Danilack [23]	Levine [24]	Mazouni [25]	Rossi [26]	Sovio [27]	Wie [28]	Zhou [29]
Bishops score			✕					
Maternal BMI	✕		✕			✕		
Estimated fetal weight						✕	✕	✕
Excessive fetal growth		✕						
Femur length							✕	
Fetal abdominal circumference	✕						✕	✕
Fetal head circumference	✕							
Fibroids		✕						
Gestational age		✕	✕		✕		✕	
Maternal height	✕		✕	✕	✕	✕	✕	✕
History of Herpes		✕						
History of previous delivery				✕	✕			
Indication for induction of labour								✕
Initial cervical consistency								✕
Initial cervical effacement								✕
Initial station								✕
Maternal age	✕	✕		✕	✕	✕	✕	✕
Maternal race		✕			✕			
Maternal weight gain						✕		
Obesity		✕						
Parity		✕	✕	✕				
Pregnancy‐associated hypertension							✕	
Uterine height								✕
Maternal weight at delivery					✕		✕	

Several research studies have tried to determine the optimal way to present predictions [30, 31]. By utilising recommendations, we determined if a model would be immediately clinically useful without any prior or further knowledge beyond the paper.

Among the 56 developed models identified, 21 provided user‐friendly tools, for example nomograms or applications to predict risk, enhancing accessibility. Of the eight low risk and externally validated models, four [22, 24, 25, 28] presented the model using a nomogram, one study created a webpage/application and another presented as an interactive Excel file [26]. Only these six studies presented models immediately useful to clinicians and thus could be considered for immediate use in a clinical setting. The two remaining studies expressed their model results in terms of their regression coefficients [23, 27].These eight low‐risk models were made in the United Kingdom (*n* = 1), the United States (*n* = 3), France (*n* = 1), China (*n* = 1), The Republic of Korea (*n* = 1) and Ireland (*n* = 1), between 2008 and 2023. These models were made at a single time point, or using data from multiple time points and have not been recalibrated. All eight studies achieved good C‐statistics (above 0.67); the two highest achieved a C‐statistic of 0.82 [23] and 0.73 [24], which is extremely good. Notably, both models are tailored for induced labour scenarios.

A table detailing all characteristics for these eight low‐risk studies can be found below (Table 4).

**TABLE 4:** Table of characteristics studies assessed as having low risk of bias.

Study	Population	Sample Size	Indication	Model intended for	Method	Presentation	C‐Statistic of externally validated model
Burke [22]	Nulliparous	2336	Induced or spontaneous	39–40 weeks	Logistic	Nomogram	0.72
Danilack [23]	Multip/Nullip	17 370	Induced	Pre‐induction	Logistic	Regression coefficients	0.82
Levine [24]	Multip/Nullip	491	Induced	Pre‐induction	Logistic	Nomogram	0.73
Mazouni [25]	Multip/Nullip	246	Induced	Antenatally	Logistic	Nomogram	0.88
Rossi [26]	Multip/Nullip	4 177 644	Induced	Pre‐induction	Logistic	Webpage/App	0.78
Sovio [27]	Nulliparous	3047	Induced or spontaneous	36 weeks	Logistic	Excel File / calculator	0.71
Wie [28]	Nulliparous	4584	Induced or spontaneous	Before onset of labour	Logistic	Regression coefficients	0.69
Zhou [29]	Nulliparous	2950	Inducted	During induction	Logistic	Nomogram	0.67

## 4 Discussion

### 4.1 Main Findings

This systematic review provides an up‐to‐date summary of existing prognostic prediction models predicting the risk of an EmCBs. The review identified 63 relevant prediction modelling studies considering the risk of EmCBs. Maternal age, maternal height, parity and gestational age were the most frequently occurring predictors. Half of these studies (33/63) were graded low risk of bias, and of these eight underwent external validation, demonstrating their predictive accuracy in an independent dataset, and are suitable to be considered for use in a clinical setting. Of these eight, six studies presented their model in a way that is immediately useful to clinicians.

### 4.2 Strengths and Limitation

The review critically appraised the 63 identified prediction modelling studies using the PROBAST tool which enabled a focused and transparent approach to assessing the risk of bias among prediction models [32]. The review contains non‐English studies thus reducing the bias introduced through restricting to English.

However, our systematic review considered clinical prediction models developed using any methodology. Twelve (out of 63) studies were built using machine learning, and we ascribed a moderate to high risk of bias to these due to lacking information to conclude risk of bias according to the PROBAST tool, which requires transparent reporting of all elements of prediction model development and therefore, the machine learning elements, by their nature, are difficult to classify as low risk [33]. PROBAST‐AI is currently under development to provide guidance on relevant methodological aspects of models developed using any AI or machine learning methods [34].

The PROBAST tool was developed in 2019 to provide researchers, reviewers and developers statistical guidance in assessing models risk of bias and applicability concerns [32]. Since the ever‐growing increase of prediction models in the recent years, some guidance is now out‐dated. For example, it is now recognised that EPV is not the optimal method of dictating sample size calculations for prediction models. There has not been an updated version of PROBAST, yet.

The review also highlighted frequently occurring predictors, but it is essential to note that frequency does not necessarily imply accuracy alone. These predictors are typically routinely collected during consultations. Including predictors that are not commonly collected could be impractical for a model, as it could compromise feasibility and limit its applications in clinical practice.

### 4.3 Interpretation

A prediction model relies on well‐conducted and reported external validation to ensure that predictions are reliable and applicable to the population using the model, for example by ensuring diversity in terms of geography, maternal complications and ethnic backgrounds. This review specifically evaluated the methodological approach and found that most studies describing some form of validation were poorly reported with key details frequently not presented. Whilst researchers are encouraged to use the guidelines, such as the Transparent Reporting of a multivariable prediction model for Individual Prognosis or Diagnosis (TRIPOD), when publishing their work, efforts are needed to encourage journal editors to make submission of a TRIPOD checklist compulsory, akin to CONSORT checklists for randomised controlled trials [35].

We have illustrated this need with this review, as within the last 5 years there has been a steady increase in the development of prediction models; however, many (30/63) of the prediction models reported in this review did not meet the defined quality standards for conduct. This renders them as yet insufficiently developed for implementation into clinical care in any setting. Taking the United Kingdom as an example, five of all 63 models [26, 36, 37, 38, 39] developed within the United Kingdom; three of the studies focused on EmCBs for women who were induced [37, 38, 39]; two focused on nulliparous women [26, 37] and three included a mixed population of nulliparous and multiparous women [36, 38, 39]. Of these models, three were internally [37, 38, 39], one externally validated [26] and all four presented easily accessible models.

The varying study populations highlight the necessity for tailored clinical prediction models. Whilst creating these models is feasible, they should be informed by clinical guidelines to ensure their applicability to the appropriate groups. For instance, it may be more effective to develop separate models for nulliparous and multiparous women rather than combining different clinical populations. Similarly, distinct models might be more suitable for low‐risk and high‐risk populations of women.

Remaining with the UK example (data in the Table S4 files for the United States, China, The Republic of Korea, Ireland and France; where the other clinically relevant prediction models are based) only one is clinically useful and externally validated [26]. This model has been robustly validated from a cohort from Cambridge (UK), with data spanning from 2008 to 2012 and externally validated using a large Scottish dataset from 2003 to 2008. The model focused on ‘low‐risk’ pregnancies, defined as women with no pre‐existing medical conditions and/or pregnancy complications and received a c‐statistic of 0.71 in both data samples, an acceptable discrimination with little evidence of overfitting. However, although The Pregnancy Outcome Prediction POPS study developed a model that is accurate and validated, the increase in prevalence of emergency caesarean birth since 2008 points towards the need to re‐evaluate predictors of EmCB in the United Kingdom [8].

Developing and internally/externally validating models is not the end of the journey for a prediction modelling. Models are context specific, and the coefficients are likely to evolve over time with changes in practice. For this reason, they require regular updates to the model to ensure they continue to perform appropriately [40]. Most commonly, and statistically advised, this is done through recalibration and adjustments of the model's intercept and/or predictor coefficients. Sometimes, recalibration is not feasible, due to dramatic changes in significant predictors or poor performance in external populations, and a new model should be developed. Continuing with the UK example, only one [36] model was developed during or, after 2018, with no evidence of recalibration since then. In fact, across the entire review, 12 were developed on data after 2018 and none of the prediction models had been re‐visited for recalibration.

However, aside from the statistical quality of a model, to be implementable in clinical practice, the models need not only to be externally validated and in a readily accessible format for clinicians or mothers to use, but also provide clinicians and women with the information to support their clinical conversations and decision making in good time. The review highlighted six models meeting these first two criteria but, of the six readily available models, only one provided prediction early enough, at 36 weeks [26], to facilitate these clinical conversations.

## 5 Conclusion

This systematic review offers an overview of global prediction models predicting the risk of EmCBs. Despite numerous models existing, we have identified high risk of bias in over half of them, with eight models presenting a low risk of bias and external validations. Only six can be readily used by clinicians and mothers and are therefore ready for immediate use in a clinical setting. However, even these well‐refined models have not been recalibrated since development, which brings into question their generalisability. Reassuringly, the predictors we identified as most frequent, also appeared in these six models. However, only one model developed in a relatively low‐risk population with data collected a decade ago is currently practically useful. Assessment at 36 weeks allows enough time to arrange a pCB, if desired. To improve personalised clinical conversations, there is a pressing need for a prediction model that accurately predicts a timely risk of an EmCB across diverse risk backgrounds.

## Author Contributions

A.H., A.M. and L.B. conceived the idea, designed the study and drafted the manuscript. J.H., M.L. and G.C. provided statistical expertise for the design and analysis of the study. All authors contributed to the critical analysis of the study and contributed to the final version of the manuscript.

## Conflicts of Interest

The authors report no conflict of interest.

## Supporting information


Data S1.

## Data Availability

The data that supports the findings of this study are available in the supplementary material of this article.
