# Supplementary material for: Systematic Review of Clinical Prediction Models for the Risk of Emergency Caesarean Births
Source: BJOG. 2024 Sep 10;132(3):231–40. doi: 10.1111/1471-0528.17948 (PMC11704077; doi:10.1111/1471-0528.17948)
Supplement: Supplementary file 1 — Data S1. [file BJO-132-231-s001.docx]

# Supplementary Material

S1: Search strategy used to identify relevant studies. (Medline)

1 ((Validat$.mp. or Predict$.ti. or Rule$.mp. or (Predict$ and (Outcome$ or Risk$ or Model$)).mp.) and (Predict$ or Model$ or Decision$ or Identif$ or Prognos$).mp.) or (Risk$.mp. and ((Model$ or Clinical$).mp. or Logistic Models/)) or (Prognostic and (History or Variable$ or Criteria or Scor$ or Characteristic$ or Find$ or Factor$ or Model$)).mp.

2 Cesarean.tw.

3 Caesarean.tw.

4 Cesarian.tw.

5 Caesarian.tw.

6 C-Section.tw.

7 ("C Section" or "C Sections").tw.

8 (cesarea$ or caesarea$).tw.

9 (postcesarea$ or postcaesarea$).tw.

10 Predict$.tw. or Predictive value of tests.mp.

11 exp Cesarean Section/

12 2 or 3 or 4 or 5 or 6 or 7 or 8 or 9

13 11 or 12

14 1 and 10 and 13

Figure S1: Geographical representation of the number of studies recruiting from a country.
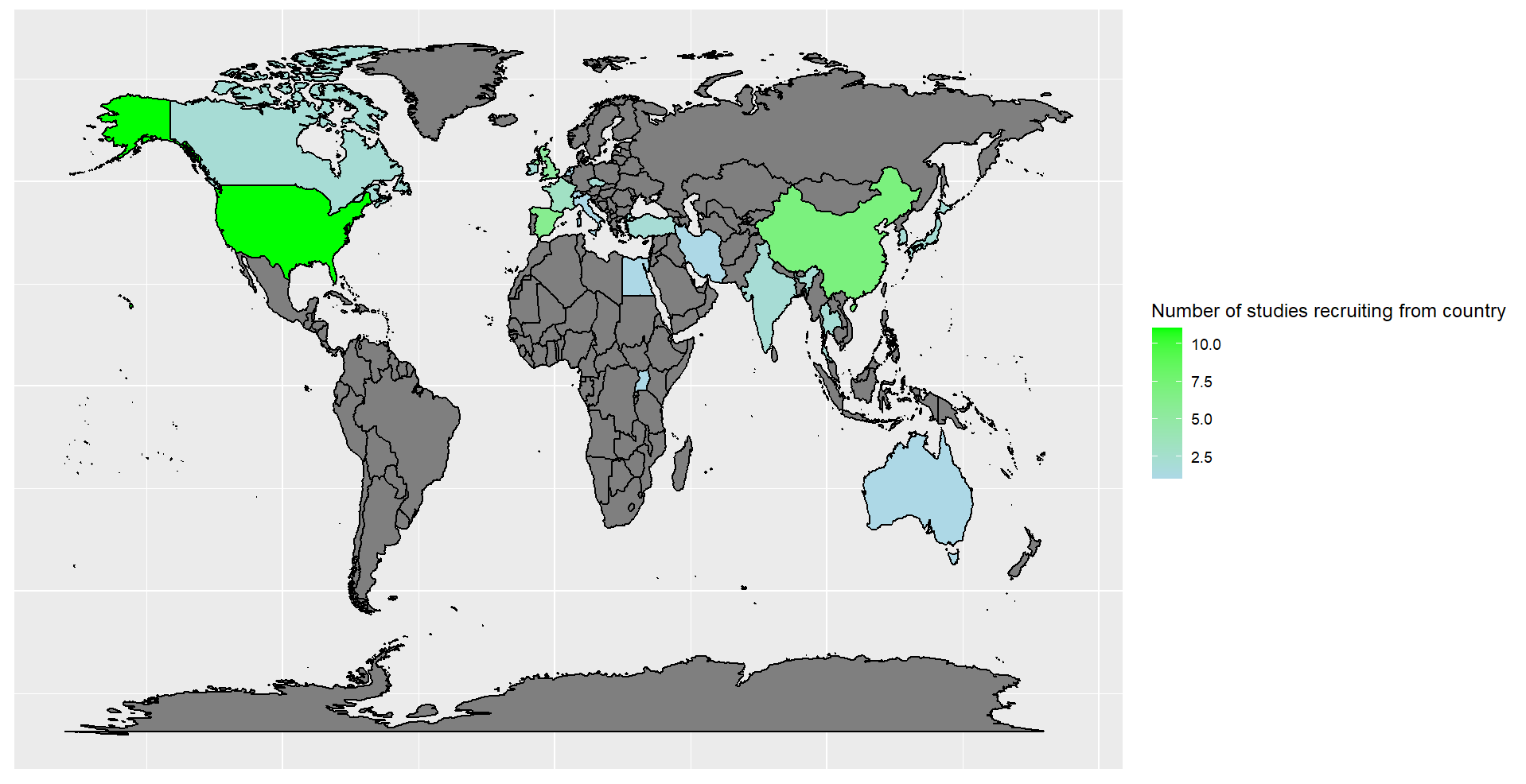


Table S1: Frequency of all predictors identified within the 63 studies.

| Maternal Height | 32 |
| --- | --- |
| Maternal age | 31 |
| Parity | 26 |
| BMI | 22 |
| Gestational age | 18 |
| Bishop score | 9 |
| Previous Caesarean section | 9 |
| Cervical Dilatation | 7 |
| Estimated fetal weight | 7 |
| Maternal weight | 7 |
| Fetal AC | 6 |
| Maternal weight gain | 6 |
| New born weight | 6 |
| Station | 6 |
| Indication for induction | 6 |
| Cervical Length | 5 |
| Fetal gender | 5 |
| Maternal race | 5 |
| Previous vaginal birth | 5 |
| Synthesis fundal height region | 4 |
| Hypertension | 4 |
| Pre-existing diabetes | 4 |
| Oxytocin use | 3 |
| Type of induction | 3 |
| Effacement | 2 |
| Epidural | 2 |
| Head progression on distance at 2 hours | 2 |
| Macrosomia | 2 |
| Mode of onset | 2 |
| Occiput posterior position | 2 |
| Post-date pregnancy | 2 |
| Prolonged latent phase | 2 |
| PRoM | 2 |
| SFH | 2 |
| Smoker | 2 |
| Progression on angle | 2 |
| Other risk factors | 2 |
| Comorbidity | 2 |
| Gestational diabetes | 2 |
| Number of previous CS | 2 |
| Obstetric risk | 2 |
| Cervix position | 1 |
| Amniotic fluid contamination | 1 |
| Abnormal position | 1 |
| AFI | 1 |
| Amniotic Membrane | 1 |
| Arrest of labour | 1 |
| Bilateral femoral head distance | 1 |
| Blood pressure | 1 |
| BPD | 1 |
| Cervical consistency | 1 |
| CESD score | 1 |
| CPR | 1 |
| Degree composition | 1 |
| Delivery time | 1 |
| Doctor | 1 |
| Doctor CS rate | 1 |
| Drug abuse | 1 |
| Excessive fetal growth | 1 |
| Fasting | 1 |
| Femur length | 1 |
| Fetal presentation | 1 |
| Fetal distress | 1 |
| Fetal head perineal distance | 1 |
| Fetal movements | 1 |
| Fetal indication | 1 |
| Fever in labour | 1 |
| Fibroids | 1 |
| Gravida | 1 |
| Haemoglobin | 1 |
| HBA1C | 1 |
| Head to synthesis distance at Valsalva | 1 |
| Heart status | 1 |
| Active Herpes | 1 |
| Engagement during Active Phase | 1 |
| History of herpes | 1 |
| Hospital | 1 |
| Hospital CS rate | 1 |
| Irregular contractions | 1 |
| Liquor appearance | 1 |
| Maternal indication | 1 |
| Maternal heart rate | 1 |
| Maternal disorders | 1 |
| Maternal fetal ratio | 1 |
| Meconium staining | 1 |
| Night shift delivery | 1 |
| Number of contractions in 10 mins | 1 |
| Obesity | 1 |
| Obstetric conjugate | 1 |
| Oligohydramnios | 1 |
| Pain | 1 |
| PAH | 1 |
| Perception of labour lasting > 24 hours | 1 |
| Polyhyroamnios | 1 |
| Prominent ischial spines | 1 |
| Placenta abruption | 1 |
| Placenta previa | 1 |
| Preeclampsia | 1 |
| Previous preeclampsia | 1 |
| Pre-pregnancy hypertension | 1 |
| Regional anesthesia | 1 |
| Scalp pH | 1 |
| SBP | 1 |
| Time between admission and 4cm | 1 |
| Fertility treatment | 1 |
| Induction time | 1 |
| Uterine contractions | 1 |
| Uterine height | 1 |
| Bridged race Black | 1 |
| Hispanic origin mexican | 1 |
| Hispanic origin Cuban | 1 |
| Bridges race Asian Indian | 1 |
| Hispanic origin non-hispanic | 1 |
| Hispanic origin portorican | 1 |
| Hispanic origin central/south american | 1 |
| Bridges race chinese | 1 |
| Bridged race white | 1 |
| bridges race other asian | 1 |
| ART mode | 1 |
| ART | 1 |
| Amniocentitis | 1 |

Table S2: The risk of bias assessment results of the included studies across the four PROBAST domains. These domains determine the overall risk of bias per study.

| **Level of bias** | **Participant Selection** | **Predictor** | **Outcome** | **Analysis** | **Overall** |
| --- | --- | --- | --- | --- | --- |
| High risk of bias | 4 | 13 | 0 | 2 | 33 |
| Low risk of bias | 59 | 50 | 63 | 61 | 30 |

Table S3: Risk of bias introduced into each domain and signalling questions. “-” indicates a high risk of bias introduced, “+” a low risk of bias, per domain and overall assessment.

| Authors | Title | Participants | Predictor | Outcome | Analysis | Overall |
| --- | --- | --- | --- | --- | --- | --- |
| Al Housseini, A.; Newman, T.; Cox, A.; Devoe, L. D. | Prediction of risk for cesarean delivery in term nulliparas: a comparison of neural network and multiple logistic regression models | + | + | + | + | - |
| Alvarez-Colomo, Cristina; Gobernado-Tejedor, Julio; Gobernado-Tejedor, Julio Alberto | The validity of ultrasonography in predicting the outcomes of labour induction | + | + | + | + | - |
| Alzola, I.; Murua, E.; Rodriguez, J.; Burgos, J.; Maiz, N. | Can the Progression Angle before Labor Help to Predict Cesarean Section? | + | + | + | + | + |
| Bademkiran, M. H.; Bademkiran, C.; Ege, S.; Peker, N.; Sucu, S.; Obut, M.; Demirel, M. O.; Samanci, S.; Bagli, I.; Celik, K. | Explanatory variables and nomogram of a clinical prediction model to estimate the risk of caesarean section after term induction | + | - | + | + | - |
| Beksac, M. S.; Tanacan, A.; Bacak, H. O.; Leblebicioglu, K. | Computerized prediction system for the route of delivery (vaginal birth versus cesarean section) | + | - | + | + | - |
| Bertossa, P.; Novakov Mikic, A.; Stupar, Z. T.; Milatovic, S.; Boulvain, M.; Irion, O.; de Tejada, B. | Validity of clinical and ultrasound variables to predict the risk of cesarean delivery after induction of labor | + | + | + | + | + |
| Branger, B.; Dochez, V.; Gervier, S.; Winer, N. | [Cesarean after labor induction: Risk factors and prediction score] | - | + | + | + | - |
| Burke, N.; Burke, G.; Breathnach, F.; McAuliffe, F.; Morrison, J. J.; Turner, M.; Dornan, S.; Higgins, J. R.; Cotter, A.; Geary, M.; McParland, P.; Daly, S.; Cody, F.; Dicker, P.; Tully, E.; Malone, F. D. | Prediction of cesarean delivery in the term nulliparous woman: results from the prospective, multicenter Genesis study | + | + | + | + | + |
| Campillo-Artero, C.; Serra-Burriel, M.; Calvo-Perez, A. | Predictive modeling of emergency cesarean delivery | + | + | + | + | + |
| Chen, Cheng; Yang, Mengmeng; Zheng, Weizeng; Yang, Xiaofu; Chen, Yuan; Dong, Tian; Lv, Min; Xi, Fangfang; Jiang, Ying; Ying, Xia; Li, Wen; Xu, Jian; Zhao, Baihui; Luo, Qiong | Magnetic Resonance Imagingâ€Based Nomogram to Antenatal Predict Cesarean Delivery for Cephalopelvic Disproportion in Primiparous Women | + | + | + | + | + |
| Chor, C. M.; Poon, L. C. Y.; Leung, T. Y. | Prediction of labor outcome using serial transperineal ultrasound in the first stage of labor | + | + | + | + | - |
| Danilack, V. A.; Danilack, V. A.; Danilack, V. A.; Hutcheon, J. A.; Triche, E. W.; Dore, D. D.; Dore, D. D.; Muri, J. H.; Phipps, M. G.; Phipps, M. G.; Phipps, M. G.; Savitz, D. A.; Savitz, D. A.; Savitz, D. A. | Development and Validation of a Risk Prediction Model for Cesarean Delivery after Labor Induction | + | + | + | + | + |
| De Ramon Fernandez, A.; Ruiz Fernandez, D.; Prieto Sanchez, M. T. | Prediction of the mode of delivery using artificial intelligence algorithms | + | - | + | + | - |
| de Souza, H. C. C.; Perdona, G. S. C.; Marcolin, A. C.; Oyeneyin, L. O.; Oladapo, O. T.; Mugerwa, K.; Souza, J. P. | Development of caesarean section prediction models: secondary analysis of a prospective cohort study in two sub-Saharan African countries | + | + | + | + | + |
| Dorwal, M.; Yadav, G.; Singh, P.; Kathuria, P.; Gothwal, M.; Ghuman, N. K.; Shekhar, S. | Deriving a prediction model for emergency cesarean delivery following induction of labor in singleton term pregnancies | + | + | + | + | - |
| Fergus, Paul; Hussain, Abir; Al-Jumeily, Dhiya; De-Shuang, Huang; Bouguila, Nizar; Huang, De-Shuang | Classification of caesarean section and normal vaginal deliveries using foetal heart rate signals and advanced machine learning algorithms | + | - | + | + | - |
| Flatley, C.; Gibbons, K. S.; Hurst, C.; Kumar, S. | Development of a cross-validated model for predicting emergency cesarean for intrapartum fetal compromise at term | + | + | + | + | + |
| Furuita, N.; Okabayashi, S.; Yamanishi, A.; Nakamura, M.; Kawamura, T. | The development of a prediction model for arrest of labour to be used at regular check-ups, during 36 or 37 gestational weeks, for primiparas: a retrospective cohort study | + | + | + | + | + |
| Guan, P.; Tang, F.; Sun, G.; Ren, W. | Prediction of emergency cesarean section by measurable maternal and fetal characteristics | + | + | + | + | - |
| Guedalia, J.; Lipschuetz, M.; Cohen, S. M.; Sompolinsky, Y.; Walfisch, A.; Sheiner, E.; Sergienko, R.; Rosenbloom, J.; Unger, R.; Yagel, S.; Hochler, H. | Transporting an Artificial Intelligence Model to Predict Emergency Cesarean Delivery: Overcoming Challenges Posed by Interfacility Variation | + | - | + | - | - |
| Harper, D. M.; Johnson, C. A.; Harper, W. H.; Liese, B. S. | Prenatal predictors of cesarean section due to labor arrest | + | + | + | + | + |
| Harper, L. M.; Odibo, A. O.; Macones, G. A.; Cahill, A. G. | Predicting cesarean in the second stage of labor | + | + | + | + | + |
| Hemmatzadeh, Shahla; Abbasalizadeh, Fatemeh; Mohammad-Alizadeh-Charandabi, Sakineh; Jafarabadi, Mohammad Asghari; Mirghafourvand, Mojgan | Development and Validation of a Nomogram to Estimate Risk of Cesarean After Induction of Labor in Term Pregnancies with an Unfavorable Cervix in Iran | + | - | + | + | - |
| HernÃ¡ndez-MartÃ­nez, Antonio; Pascual-PedreÃ±o, Ana; BaÃ±o-GarnÃ©s, Ana; Melero-JimÃ©nez, MarÃ­a; TenÃ­as-Burillo, JosÃ©; Molina-AlarcÃ³n, Milagros; HernÃ¡ndez-MartÃ­nez, Antonio; Pascual-PedreÃ±o, Ana I.; BaÃ±o-GarnÃ©s, Ana B.; Melero-JimÃ©nez, MarÃ­a R.; TenÃ­as-Burillo, JosÃ© M.; Molina-AlarcÃ³n, Milagros | Predictive model for risk of cesarean section in pregnant women after induction of labor | + | + | + | + | + |
| Hin, L. Y.; Lau, T. K.; Rogers, M. S.; Chang, A. M. Z. | Dichotomization of continuous measurements using generalized additive modelling application in predicting intrapartum caesarean delivery | + | + | + | + | - |
| Hin, L. Y.; Lau, T. K.; Rogers, M.; Chang, A. M. Z. | Antepartum and intrapartum prediction of cesarean need: Risk scoring in singleton pregnancies | + | + | + | + | - |
| Hueston, W. J. | Development of a cesarean delivery risk score | + | + | + | + | - |
| Isono, W.; Nagamatsu, T.; Uemura, Y.; Fujii, T.; Hyodo, H.; Yamashita, T.; Kamei, Y.; Kozuma, S.; Taketani, Y. | Prediction model for the incidence of emergent cesarean section during induction of labor specialized in nulliparous low-risk women | + | - | + | + | - |
| Janssen, P. A.; Stienen, J. J. C.; Brant, R.; Hanley, G. E. | A Predictive Model for Cesarean Among Low-Risk Nulliparous Women in Spontaneous Labor at Hospital Admission | + | + | + | + | + |
| Jochum, Floriane; Le Ray, Camille; Severac, FranÃ§ois; Sananes, Nicolas | Externally Validated Score to Predict Cesarean Delivery After Labor Induction With Cervical Ripening | + | + | + | + | + |
| Kamel, R. A.; Negm, S. M.; Youssef, A.; Bianchini, L.; Brunelli, E.; Pilu, G.; Soliman, M.; Nicolaides, K. H. | Predicting cesarean delivery for failure to progress as an outcome of labor induction in term singleton pregnancy | + | + | + | + | + |
| Khan, N. I.; Mahmud, T.; Islam, M. N.; Mustafina, S. N. | Prediction of Cesarean Childbirth using Ensemble Machine Learning Methods | + | - | + | - | - |
| Khunpradit, S.; Patumanond, J.; Tawichasri, C. | Validation of risk scoring scheme for cesarean delivery due to cephalopelvic disproportion in Lamphun Hospital | - | + | + | + | - |
| Khunpradit, S.; Patumanond, J.; Tawichasri, C. | Development of risk scoring scheme for prediction of cesarean delivery due to cephalopelvic disproportion in Lamphun Hospital, Thailand | - | + | + | + | - |
| Kim, S. N.; Park, K. H.; Jung, H. J.; Hong, J. S.; Shin, D. M.; Kang, W. S.; Kim, S. N.; Park, K. H.; Jung, H. J.; Hong, J. S.; Shin, D. M.; Kang, W. S. | Clinical and sonographic parameters at 37 weeks' gestation for predicting the risk of primary Cesarean delivery in nulliparous women | + | + | + | + | + |
| Krepelka, P.; Urbankova, I.; Krofta, L.; Hanacek, J.; Feyereisl, J. | A model for predicting unscheduled caesarean section in nulliparae | + | + | + | + | + |
| Levine, L. D.; Downes, K. L.; Parry, S.; Elovitz, M. A.; Sammel, M. D.; Srinivas, S. K. | A validated calculator to estimate risk of cesarean after an induction of labor with an unfavorable cervix | + | + | + | + | + |
| Lu, H.; Hirst, J.; Yang, J.; Mackillop, L.; Clifton, D. | Standardising the assessment of caesarean birth using an oxford caesarean prediction score for mothers with gestational diabetes | + | + | + | + | - |
| Luthy, D. A.; Malmgren, J. A.; Zingheim, R. W.; Leininger, C. J.; Luthy, David A.; Malmgren, Judith A.; Zingheim, Rosalee W.; Leininger, Christopher J. | Physician contribution to a cesarean delivery risk model | + | + | + | + | - |
| Mazouni, C.; Rouzier, R.; Collette, E.; Menard, J. P.; Magnin, G.; Gamerre, M.; Deter, R. | Development and validation of a nomogram to predict the risk of cesarean delivery in macrosomia | + | + | + | + | + |
| Meyer, R.; Weisz, B.; Eilenberg, R.; Tsadok, M. A.; Uziel, M.; Sivan, E.; Mazaki-Tovi, S.; Tsur, A. | Utilizing machine learning to predict unplanned cesarean delivery | + | + | + | + | + |
| Migliorelli, F.; Banos, N.; Angeles, M. A.; Rueda, C.; Salazar, L.; Gratacos, E.; Palacio, M. | Clinical and Sonographic Model to Predict Cesarean Delivery after Induction of Labor at Term | + | + | + | + | + |
| Murphy, N. C.; Burke, N.; Dicker, P.; Cody, F.; Nafisee, S. A.; Deleau, D.; Kent, E.; Ramaiah, S.; Tully, E. C.; Malone, F. D.; Breathnach, F. M. | Reducing emergency cesarean delivery and improving the primiparous experience: Findings of the RECIPE study | + | + | + | + | + |
| Nwabuobi, C.; Gowda, N.; Schmitz, J.; Wood, N.; Pargas, A.; Bagiardi, L.; Odibo, L.; Camisascaâ€Lopina, H.; Kuznicki, M.; Sinkey, R.; Odibo, A.; Camisasca-Lopina, H. | Risk factors for Cesarean delivery in pregnancy with small-for-gestational-age fetus undergoing induction of labor | + | + | + | + | + |
| Paidas Teefey, C.; Reforma, L.; Koelper, N. C.; Sammel, M. D.; Srinivas, S. K.; Levine, L. D.; Durnwald, C. P. | Risk Factors Associated With Cesarean Delivery After Induction of Labor in Women With Class III Obesity | + | + | + | + | + |
| Papoutsis, D.; Antonakou, A.; Gornall, A.; Tzavara, C.; Mohajer, M. | The SaTH risk-assessment tool for the prediction of emergency cesarean section in women having induction of labor for all indications: a large-cohort based study | + | + | + | + | + |
| Peregrine, E.; Oâ€™Brien, P.; Omar, R.; Jauniaux, E. | Clinical and ultrasound parameters to predict the risk of cesarean delivery after induction of labor | + | + | + | + | + |
| Rossi, R. M.; Requarth, E.; Warshak, C. R.; Dufendach, K. R.; Hall, E. S.; DeFranco, E. A. | Risk Calculator to Predict Cesarean Delivery Among Women Undergoing Induction of Labor | + | + | + | + | + |
| Seshadri, L.; Mukherjee, B. | A predictive model for cesarean section in low risk pregnancies | + | + | + | + | - |
| Smith, G. C. S.; Dellens, M.; White, I. R.; Pell, J. P. | Combined logistic and Bayesian modeling of cesarean section risk | + | + | + | + | - |
| Sodsee, S. | Predicting caesarean section by applying nearest neighbor analysis | + | + | + | + | - |
| Sovio, U.; Smith, G. C. S. | Blinded ultrasound fetal biometry at 36â€‰weeks and risk of emergency Cesarean delivery in a prospective cohort study of low-risk nulliparous women | + | + | + | + | + |
| Sun, F.; Shen, M.; Han, B.; Chen, Y.; Wu, F. | Validation of three models (Tolcher, Levine, and Burke) for predicting term cesarean section in Chinese population | + | + | + | + | + |
| Sun, F.; Wu, F.; Shen, M.; Han, B. | A model for predicting cesarean delivery in term low-risk primipara after failure of trial of labor | + | - | + | + | - |
| Tolcher, M. C.; Holbert, M. R.; Weaver, A. L.; McGree, M. E.; Olson, J. E.; El-Nashar, S. A.; Famuyide, A. O.; Brost, B. C. | Predicting Cesarean Delivery After Induction of Labor Among Nulliparous Women at Term | + | + | + | + | + |
| Tun, M. H.; Chari, R.; Kaul, P.; Mamede, F. V.; Paulden, M.; Lefebvre, D. L.; Turvey, S. E.; Moraes, T. J.; Sears, M. R.; Subbarao, P.; Mandhane, P. J. | Prediction of odds for emergency cesarean section: A secondary analysis of the CHILD term birth cohort study | + | + | + | + | - |
| Verhoeven, C. J. M.; Oudenaarden, A.; Hermus, M. A. A.; Porath, M. M.; Oei, S. G.; Mol, B. W. J. | Validation of models that predict Cesarean section after induction of labor | + | - | + | + | - |
| Wie, J. H.; Lee, S. J.; Choi, S. K.; Jo, Y. S.; Hwang, H. S.; Park, M. H.; Kim, Y. H.; Shin, J. E.; Kil, K. C.; Kim, S. M.; Choi, B. S.; Hong, H.; Seol, H. J.; Won, H. S.; Ko, H. S.; Na, S. | Prediction of Emergency Cesarean Section Using Machine Learning Methods: Development and External Validation of a Nationwide Multicenter Dataset in Republic of Korea | + | + | + | + | + |
| Wu, Chiung-Hui; Chen, Chiu-Fen; Chien, Chi-Chen | Prediction of dystocia-related cesarean section risk in uncomplicated Taiwanese nulliparas at term | + | + | + | + | + |
| Yang, Y. S.; Hur, M. H.; Kim, S. Y. | Risk factors of cesarean delivery at prenatal care, admission and during labor in low-risk pregnancy: multivariate logistic regression analysis | + | - | + | + | - |
| Yang, YunSeok | An intrapartum calculator for predicting cesarean birth due to dystocia: Preliminary findings from a singleâ€center study in Korea | + | - | + | + | - |
| Yoko, Nagayasu; Daisuke, Fujita; Masahide, Ohmichi; Yoichi, Hayashi | Use of an artificial intelligence-based rule extraction approach to predict an emergency cesarean section | + | - | + | + | - |
| Zhou, H.; Gu, N.; Yang, Y.; Wang, Z.; Hu, Y.; Dai, Y. | Nomogram predicting cesarean delivery undergoing induction of labor among high-risk nulliparous women at term: a retrospective study | + | + | + | + | + |

Table S4: Table of characteristics for all 63 studies included within the review.

| Authors | Title | Year of publication | Country (of data/recruitment) | Study  Design | Nulliparous/multiparous women | Type of labour | Total sample size population (prediction model) | Outcome | External Validation |
| --- | --- | --- | --- | --- | --- | --- | --- | --- | --- |
| Al Housseini, A.; Newman, T.; Cox, A.; Devoe, L. D. | Prediction of risk for cesarean delivery in term nulliparas: a comparison of neural network and multiple logistic regression models | 2009 | USA | Retrospective cohort | Nulliparous | Mixed | 700 | EmCS | no |
| Alvarez-Colomo, Cristina; Gobernado-Tejedor, Julio; Gobernado-Tejedor, Julio Alberto | The validity of ultrasonography in predicting the outcomes of labour induction | 2016 | Spain | Prospective Observational study | Mixed | Induced | 151 | EmCS | no |
| Alzola, I.; Murua, E.; Rodriguez, J.; Burgos, J.; Maiz, N. | Can the Progression Angle before Labor Help to Predict Cesarean Section? | 2020 | Spain | Prospective Observational study | Mixed | Mixed | 575 | EmCs | no |
| Bademkiran, M. H.; Bademkiran, C.; Ege, S.; Peker, N.; Sucu, S.; Obut, M.; Demirel, M. O.; Samanci, S.; Bagli, I.; Celik, K. | Explanatory variables and nomogram of a clinical prediction model to estimate the risk of caesarean section after term induction | 2021 | Turkey | Retrospective cohort | Mixed | Induced | 1328 | EmCS | no |
| Beksac, M. S.; Tanacan, A.; Bacak, H. O.; Leblebicioglu, K. | Computerized prediction system for the route of delivery (vaginal birth versus cesarean section) | 2018 | Turkey | Retrospective cohort | Mixed | Mixed | 4451 | EmCS | no |
| Bertossa, P.; Novakov Mikic, A.; Stupar, Z. T.; Milatovic, S.; Boulvain, M.; Irion, O.; de Tejada, B. | Validity of clinical and ultrasound variables to predict the risk of cesarean delivery after induction of labor | 2012 | Switzerland & Serbia | Prospective Observational study | Mixed | Induced | 537 | EmCS | Validation study |
| Branger, B.; Dochez, V.; Gervier, S.; Winer, N. | [Cesarean after labor induction: Risk factors and prediction score] | 2018 | France | Retrospective case control study | Mixed | Induced | 941 | EmCS | no |
| Burke, N.; Burke, G.; Breathnach, F.; McAuliffe, F.; Morrison, J. J.; Turner, M.; Dornan, S.; Higgins, J. R.; Cotter, A.; Geary, M.; McParland, P.; Daly, S.; Cody, F.; Dicker, P.; Tully, E.; Malone, F. D. | Prediction of cesarean delivery in the term nulliparous woman: results from the prospective, multicenter Genesis study | 2017 | Ireland | Prospective Observational study | Nulliparous | Mixed | 2336 | EmCS | yes |
| Campillo-Artero, C.; Serra-Burriel, M.; Calvo-Perez, A. | Predictive modeling of emergency cesarean delivery | 2018 | Spain | Prospective Observational study | Mixed | Mixed | 6157 | EmCS | no |
| Chen, Cheng; Yang, Mengmeng; Zheng, Weizeng; Yang, Xiaofu; Chen, Yuan; Dong, Tian; Lv, Min; Xi, Fangfang; Jiang, Ying; Ying, Xia; Li, Wen; Xu, Jian; Zhao, Baihui; Luo, Qiong | Magnetic Resonance Imagingâ€Based Nomogram to Antenatal Predict Cesarean Delivery for Cephalopelvic Disproportion in Primiparous Women | 2022 | China | Prospective Observational study | Nulliparous | Mixed | 150 | EmCS | no |
| Chor, C. M.; Poon, L. C. Y.; Leung, T. Y. | Prediction of labor outcome using serial transperineal ultrasound in the first stage of labor | 2019 | China | Prospective Observational study | Nulliparous | Mixed | 124 | EmCS for non progression | no |
| Danilack, V. A.; Danilack, V. A.; Danilack, V. A.; Hutcheon, J. A.; Triche, E. W.; Dore, D. D.; Dore, D. D.; Muri, J. H.; Phipps, M. G.; Phipps, M. G.; Phipps, M. G.; Savitz, D. A.; Savitz, D. A.; Savitz, D. A. | Development and Validation of a Risk Prediction Model for Cesarean Delivery after Labor Induction | 2020 | USA | Retrospective cohort | Mixed | Induced | 17370 | EmCS | yes |
| De Ramon Fernandez, A.; Ruiz Fernandez, D.; Prieto Sanchez, M. T. | Prediction of the mode of delivery using artificial intelligence algorithms | 2022 | Spain | Retrospective cohort | Mixed | Mixed | 11460 | EmCS | no |
| de Souza, H. C. C.; Perdona, G. S. C.; Marcolin, A. C.; Oyeneyin, L. O.; Oladapo, O. T.; Mugerwa, K.; Souza, J. P. | Development of caesarean section prediction models: secondary analysis of a prospective cohort study in two sub-Saharan African countries | 2019 | Uganda and Nigeria | Prospective Observational study | Mixed | Mixed | 5908 | EmCS | no |
| Dorwal, M.; Yadav, G.; Singh, P.; Kathuria, P.; Gothwal, M.; Ghuman, N. K.; Shekhar, S. | Deriving a prediction model for emergency cesarean delivery following induction of labor in singleton term pregnancies | 2023 | India | Prospective cross sectional study | Mixed | Induced | 454 | EmCS | no |
| Fergus, Paul; Hussain, Abir; Al-Jumeily, Dhiya; De-Shuang, Huang; Bouguila, Nizar; Huang, De-Shuang | Classification of caesarean section and normal vaginal deliveries using foetal heart rate signals and advanced machine learning algorithms | 2017 | Czech Republic | Retrospective cohort | Mixed | Mixed | 552 | EmCS | no |
| Flatley, C.; Gibbons, K. S.; Hurst, C.; Kumar, S. | Development of a cross-validated model for predicting emergency cesarean for intrapartum fetal compromise at term | 2020 | Australia | Prospective Observational study | Mixed | Mixed | 5439 | EmCS for fetal distress | no |
| Furuita, N.; Okabayashi, S.; Yamanishi, A.; Nakamura, M.; Kawamura, T. | The development of a prediction model for arrest of labour to be used at regular check-ups, during 36 or 37 gestational weeks, for primiparas: a retrospective cohort study | 2022 | Japan | Retrospective cohort | Nulliparous | Mixed | 739 | EmCS for arrest of labour | no |
| Guan, P.; Tang, F.; Sun, G.; Ren, W. | Prediction of emergency cesarean section by measurable maternal and fetal characteristics | 2020 | China | Retrospective cohort | Mixed | Mixed | 10295 | EmCS | no |
| Guedalia, J.; Lipschuetz, M.; Cohen, S. M.; Sompolinsky, Y.; Walfisch, A.; Sheiner, E.; Sergienko, R.; Rosenbloom, J.; Unger, R.; Yagel, S.; Hochler, H. | Transporting an Artificial Intelligence Model to Predict Emergency Cesarean Delivery: Overcoming Challenges Posed by Interfacility Variation | 2021 | Isreal | Retrospective cohort | Mixed | Mixed | 61012 | EmCS | yes |
| Harper, D. M.; Johnson, C. A.; Harper, W. H.; Liese, B. S. | Prenatal predictors of cesarean section due to labor arrest | 1995 | USA | Case Control | Mixed | Mixed | 361 | EmCS for arrest of labour | no |
| Harper, L. M.; Odibo, A. O.; Macones, G. A.; Cahill, A. G. | Predicting cesarean in the second stage of labor | 2013 | USA | Retrospective cohort | Mixed | Mixed | 5388 | EmCS (Second Stage) | no |
| Hemmatzadeh, Shahla; Abbasalizadeh, Fatemeh; Mohammad-Alizadeh-Charandabi, Sakineh; Jafarabadi, Mohammad Asghari; Mirghafourvand, Mojgan | Development and Validation of a Nomogram to Estimate Risk of Cesarean After Induction of Labor in Term Pregnancies with an Unfavorable Cervix in Iran | 2022 | Iran | Prospective Observational study | Mixed | Induced | 200 | EmCS | yes |
| HernÃ¡ndez-MartÃ­nez, Antonio; Pascual-PedreÃ±o, Ana; BaÃ±o-GarnÃ©s, Ana; Melero-JimÃ©nez, MarÃ­a; TenÃ­as-Burillo, JosÃ©; Molina-AlarcÃ³n, Milagros; HernÃ¡ndez-MartÃ­nez, Antonio; Pascual-PedreÃ±o, Ana I.; BaÃ±o-GarnÃ©s, Ana B.; Melero-JimÃ©nez, MarÃ­a R.; TenÃ­as-Burillo, JosÃ© M.; Molina-AlarcÃ³n, Milagros | Predictive model for risk of cesarean section in pregnant women after induction of labor | 2016 | Spain | Retrospective cohort | Mixed | Induced | 704 | EmCS | no |
| Hin, L. Y.; Lau, T. K.; Rogers, M. S.; Chang, A. M. Z. | Dichotomization of continuous measurements using generalized additive modelling application in predicting intrapartum caesarean delivery | 1999 | Hong Kong | Retrospective cohort | Mixed | unclear | 5921 | EmCS | no |
| Hin, L. Y.; Lau, T. K.; Rogers, M.; Chang, A. M. Z. | Antepartum and intrapartum prediction of cesarean need: Risk scoring in singleton pregnancies | 1997 | Hong Kong | Retrospective cohort | Mixed | Mixed | 5921 | EmCS | no |
| Hueston, W. J. | Development of a cesarean delivery risk score | 1994 | USA | Retrospective cohort | Mixed | unclear | 7517 | EmCS | no |
| Isono, W.; Nagamatsu, T.; Uemura, Y.; Fujii, T.; Hyodo, H.; Yamashita, T.; Kamei, Y.; Kozuma, S.; Taketani, Y. | Prediction model for the incidence of emergent cesarean section during induction of labor specialized in nulliparous low-risk women | 2011 | Japan | Retrospective cohort | Mixed | Induced | 392 | EmCS for PRoM | no |
| Janssen, P. A.; Stienen, J. J. C.; Brant, R.; Hanley, G. E. | A Predictive Model for Cesarean Among Low-Risk Nulliparous Women in Spontaneous Labor at Hospital Admission | 2017 | Canada | Secondary analysis of clinical trial | Nulliparous | Spontaneous | 1302 | EmCS | no |
| Jochum, Floriane; Le Ray, Camille; Severac, FranÃ§ois; Sananes, Nicolas | Externally Validated Score to Predict Cesarean Delivery After Labor Induction With Cervical Ripening | 2019 | France | Prospective Observational study | Mixed | Induced | 1024 | EmCS | Validation study |
| Kamel, R. A.; Negm, S. M.; Youssef, A.; Bianchini, L.; Brunelli, E.; Pilu, G.; Soliman, M.; Nicolaides, K. H. | Predicting cesarean delivery for failure to progress as an outcome of labor induction in term singleton pregnancy | 2021 | Egypt, Italy | Prospective Observational study | Nulliparous | Induced | 243 | EmCS for failure to progress | no |
| Khan, N. I.; Mahmud, T.; Islam, M. N.; Mustafina, S. N. | Prediction of Cesarean Childbirth using Ensemble Machine Learning Methods | 2020 | Spain | Retrospective cohort | Mixed | Mixed | 4309 | EmCS | no |
| Khunpradit, S.; Patumanond, J.; Tawichasri, C. | Validation of risk scoring scheme for cesarean delivery due to cephalopelvic disproportion in Lamphun Hospital | 2006 | Thailand | Case Control | Mixed | unclear | 526 | EmCS for CPD | Validation study |
| Khunpradit, S.; Patumanond, J.; Tawichasri, C. | Development of risk scoring scheme for prediction of cesarean delivery due to cephalopelvic disproportion in Lamphun Hospital, Thailand | 2007 | Thailand | Case Control | Mixed | Mixed | 423 | EmCS for CPD | yes |
| Kim, S. N.; Park, K. H.; Jung, H. J.; Hong, J. S.; Shin, D. M.; Kang, W. S.; Kim, S. N.; Park, K. H.; Jung, H. J.; Hong, J. S.; Shin, D. M.; Kang, W. S. | Clinical and sonographic parameters at 37 weeks' gestation for predicting the risk of primary Cesarean delivery in nulliparous women | 2010 | Korea | Prospective Observational study | Nulliparous | Mixed | 453 | EmCS | no |
| Krepelka, P.; Urbankova, I.; Krofta, L.; Hanacek, J.; Feyereisl, J. | A model for predicting unscheduled caesarean section in nulliparae | 2020 | Czech Republic | Prospective cohort study | Nulliparous | Mixed | 3728 | EmCS | no |
| Levine, L. D.; Downes, K. L.; Parry, S.; Elovitz, M. A.; Sammel, M. D.; Srinivas, S. K. | A validated calculator to estimate risk of cesarean after an induction of labor with an unfavorable cervix | 2018 | USA | Secondary analysis from RCT | Mixed | Induced | 491 | EmCS | yes |
| Lu, H.; Hirst, J.; Yang, J.; Mackillop, L.; Clifton, D. | Standardising the assessment of caesarean birth using an oxford caesarean prediction score for mothers with gestational diabetes | 2022 | UK | Prospective Observational study | Mixed | unclear | 97 | EmCS | no |
| Luthy, D. A.; Malmgren, J. A.; Zingheim, R. W.; Leininger, C. J.; Luthy, David A.; Malmgren, Judith A.; Zingheim, Rosalee W.; Leininger, Christopher J. | Physician contribution to a cesarean delivery risk model | 2003 | USA | Prospective cohort study | Mixed | Mixed | 7940 | EmCS | no |
| Mazouni, C.; Rouzier, R.; Collette, E.; Menard, J. P.; Magnin, G.; Gamerre, M.; Deter, R. | Development and validation of a nomogram to predict the risk of cesarean delivery in macrosomia | 2008 | France | Retrospective cohort | Mixed | Induced | 246 | EmCS for Macrosomia | yes |
| Meyer, R.; Weisz, B.; Eilenberg, R.; Tsadok, M. A.; Uziel, M.; Sivan, E.; Mazaki-Tovi, S.; Tsur, A. | Utilizing machine learning to predict unplanned cesarean delivery | 2022 | Isreal | Retrospective cohort | Mixed | Mixed | 73667 | EmCS | no |
| Migliorelli, F.; Banos, N.; Angeles, M. A.; Rueda, C.; Salazar, L.; Gratacos, E.; Palacio, M. | Clinical and Sonographic Model to Predict Cesarean Delivery after Induction of Labor at Term | 2019 | Spain | Prospective Observational study | Mixed | Induced | 334 | EmCS for failed induction or arrest of labour | no |
| Murphy, N. C.; Burke, N.; Dicker, P.; Cody, F.; Nafisee, S. A.; Deleau, D.; Kent, E.; Ramaiah, S.; Tully, E. C.; Malone, F. D.; Breathnach, F. M. | Reducing emergency cesarean delivery and improving the primiparous experience: Findings of the RECIPE study | 2020 | Ireland | Prospective Observational study | Nulliparous | Mixed | 559 | EmCS | Validation study |
| Nwabuobi, C.; Gowda, N.; Schmitz, J.; Wood, N.; Pargas, A.; Bagiardi, L.; Odibo, L.; Camisascaâ€Lopina, H.; Kuznicki, M.; Sinkey, R.; Odibo, A.; Camisasca-Lopina, H. | Risk factors for Cesarean delivery in pregnancy with small-for-gestational-age fetus undergoing induction of labor | 2020 | USA | Retrospective cohort | Mixed | Induced | 594 | EmCS | no |
| Paidas Teefey, C.; Reforma, L.; Koelper, N. C.; Sammel, M. D.; Srinivas, S. K.; Levine, L. D.; Durnwald, C. P. | Risk Factors Associated With Cesarean Delivery After Induction of Labor in Women With Class III Obesity | 2020 | USA | Retrospective cohort | Mixed | Induced | 594 | EmCS | Validation study |
| Papoutsis, D.; Antonakou, A.; Gornall, A.; Tzavara, C.; Mohajer, M. | The SaTH risk-assessment tool for the prediction of emergency cesarean section in women having induction of labor for all indications: a large-cohort based study | 2017 | UK | Prospective Observational study | Mixed | Induced | 6169 | EmCS | no |
| Peregrine, E.; Oâ€™Brien, P.; Omar, R.; Jauniaux, E. | Clinical and ultrasound parameters to predict the risk of cesarean delivery after induction of labor | 2006 | UK | Prospective Observational study | Mixed | Induced | 267 | EmCS | no |
| Rossi, R. M.; Requarth, E.; Warshak, C. R.; Dufendach, K. R.; Hall, E. S.; DeFranco, E. A. | Risk Calculator to Predict Cesarean Delivery Among Women Undergoing Induction of Labor | 2020 | USA | Retrospective cohort | Mixed | Induced | 4177644 | EmCS | yes |
| Seshadri, L.; Mukherjee, B. | A predictive model for cesarean section in low risk pregnancies | 2005 | India | Retrospective cohort | Mixed | Spontaneous | 987 | EmCS | yes |
| Smith, G. C. S.; Dellens, M.; White, I. R.; Pell, J. P. | Combined logistic and Bayesian modeling of cesarean section risk | 2004 | UK | Retrospective cohort | Nulliparous | Induced | 14968 | EmCS | no |
| Sodsee, S. | Predicting caesarean section by applying nearest neighbor analysis | 2014 | Thailand | unclear | Mixed | unclear | 400 | CS due to cephaloepelvic disproportion | no |
| Sovio, U.; Smith, G. C. S. | Blinded ultrasound fetal biometry at 36â€‰weeks and risk of emergency Cesarean delivery in a prospective cohort study of low-risk nulliparous women | 2018 | UK | Prospective Observational study | Nulliparous | Mixed | 3047 | EmCs | yes |
| Sun, F.; Shen, M.; Han, B.; Chen, Y.; Wu, F. | Validation of three models (Tolcher, Levine, and Burke) for predicting term cesarean section in Chinese population | 2022 | China | Retrospective cohort | Mixed | unclear | 6530467 | EmCs | Validation study |
| Sun, F.; Wu, F.; Shen, M.; Han, B. | A model for predicting cesarean delivery in term low-risk primipara after failure of trial of labor | 2020 | China | Retrospective cohort | Nulliparous | Mixed | 6551 | EmCs | yes |
| Tolcher, M. C.; Holbert, M. R.; Weaver, A. L.; McGree, M. E.; Olson, J. E.; El-Nashar, S. A.; Famuyide, A. O.; Brost, B. C. | Predicting Cesarean Delivery After Induction of Labor Among Nulliparous Women at Term | 2015 | USA | Retrospective cohort | Nulliparous | Induced | 785 | EmCs | no |
| Tun, M. H.; Chari, R.; Kaul, P.; Mamede, F. V.; Paulden, M.; Lefebvre, D. L.; Turvey, S. E.; Moraes, T. J.; Sears, M. R.; Subbarao, P.; Mandhane, P. J. | Prediction of odds for emergency cesarean section: A secondary analysis of the CHILD term birth cohort study | 2022 | Canada | Prospective Observational study | Mixed | Spontaneous | 2150 | EmCs | no |
| Verhoeven, C. J. M.; Oudenaarden, A.; Hermus, M. A. A.; Porath, M. M.; Oei, S. G.; Mol, B. W. J. | Validation of models that predict Cesarean section after induction of labor | 2009 | Netherlands | Prospective Observational study | Mixed | Induced | 240 | EmCs | Validation study |
| Wie, J. H.; Lee, S. J.; Choi, S. K.; Jo, Y. S.; Hwang, H. S.; Park, M. H.; Kim, Y. H.; Shin, J. E.; Kil, K. C.; Kim, S. M.; Choi, B. S.; Hong, H.; Seol, H. J.; Won, H. S.; Ko, H. S.; Na, S. | Prediction of Emergency Cesarean Section Using Machine Learning Methods: Development and External Validation of a Nationwide Multicenter Dataset in Republic of Korea | 2022 | South Korea | Retrospective cohort | Nulliparous | unclear | 4584 | EmCs | yes |
| Wu, Chiung-Hui; Chen, Chiu-Fen; Chien, Chi-Chen | Prediction of dystocia-related cesarean section risk in uncomplicated Taiwanese nulliparas at term | 2013 | China | Retrospective cohort | Nulliparous | Spontaneous | 1272 | EmCS related to dystocia | no |
| Yang, Y. S.; Hur, M. H.; Kim, S. Y. | Risk factors of cesarean delivery at prenatal care, admission and during labor in low-risk pregnancy: multivariate logistic regression analysis | 2013 | Korea | Retrospective cohort | Mixed | unclear | 1321 | EmCS due to failer to progress | no |
| Yang, YunSeok | An intrapartum calculator for predicting cesarean birth due to dystocia: Preliminary findings from a singleâ€center study in Korea | 2022 | South Korea | Retrospective cohort | Mixed | Mixed | 1326 | EmCS related to dystocia | no |
| Yoko, Nagayasu; Daisuke, Fujita; Masahide, Ohmichi; Yoichi, Hayashi | Use of an artificial intelligence-based rule extraction approach to predict an emergency cesarean section | 2022 | Japan | Retrospective cohort | Mixed | Mixed | 1513 | EmCs | no |
| Zhou, H.; Gu, N.; Yang, Y.; Wang, Z.; Hu, Y.; Dai, Y. | Nomogram predicting cesarean delivery undergoing induction of labor among high-risk nulliparous women at term: a retrospective study | 2022 | China | Retrospective cohort | Nulliparous | Induced | 2950 | EmCs | yes |

## Data S5: Search strategies used for CINAHL

1. SU caesarean section
2. TI ( caesarean section or cesarean section or c-section or caesarean delivery or cesarean delivery or caesarean birth or cesarean birth or birth ) OR AB ( caesarean section or cesarean section or c-section or caesarean delivery or cesarean delivery or caesarean birth or cesarean birth )
3. AB "post cesarea*" OR AB "post caesarea*"
4. TI ( c-section or cesarean section or caesarean section ) OR AB ( c-section or cesarean section or caesarean section )
5. TI Prognos* OR AB Prognos*
6. TI Predict* OR AB Predict* AND (TI Outcome* OR AB Outcome* OR TI Risk* OR AB Risk* OR TI Model* OR AB Model*)
7. S1 OR S2 OR S3 OR S4
8. S5 OR S6
9. TI Predict* OR AB Predict*
10. S7 AND S8 AND S9

## Data S6: Search strategies used for Cochrane Central

1. ((cesarea* or caesarea*)):ti,ab,kw
2. ("c section*"):ti,ab,kw
3. MeSH descriptor: [Cesarean Section] explode all trees
4. {or #1-#3}
5. (Predict* and (Outcome* or Risk* or Model*)):ti,ab,kw
6. Validat* or Predict* or Rule*:ti,ab
7. Predict* or Model* or Decision* or Identif* or Prognos*:ti,ab,kw
8. #7 or #5
9. Risk* and (Model* or Clinical* or "logistic models"):ti,ab,kw
10. #8 or #9
11. Prognostic and (History or Variable* or Criteria or Scor* or Characteristic* or Find* or Factor* or Model*)):ti,ab,kw
12. #10 or #11
13. #12 and #4 and #6

## Data S7: Search strategies used for Cochrane Central

( ( TITLE-ABS ( cesarea* OR caesarea* ) OR TITLE-ABS ( "C section" ) OR TITLE-ABS-KEY ( postcesarea* OR postcaesarea* ) ) AND ( TITLE-ABS-KEY ( predict* AND ( outcome* OR risk* OR model* ) ) OR TITLE-ABS-KEY ( validat* OR predict* OR rule* ) OR TITLE-ABS-KEY ( predict* AND model* OR decision* OR identif* OR prognos* ) ) ) AND TITLE ( predict* )
